# Cost-effectiveness analysis of dupilumab among patients with uncontrolled severe asthma using LIBERTY ASTHMA QUEST Korean data

**DOI:** 10.1186/s13561-024-00532-4

**Published:** 2024-08-26

**Authors:** Sung-Hee Oh, Chin Kook Rhee, Eun Jin Bae, Hyemin Ku

**Affiliations:** 1 BK21 FOUR Community-Based Intelligent Novel Drug Discovery Education Unit, College of Pharmacy and Research Institute of Pharmaceutical Sciences, Kyungpook National University, Daegu, 41566 Republic of Korea; 2 Division of Pulmonary and Critical Care Medicine, Department of Internal Medicine, College of Medicine, Seoul St. Mary’s Hospital, The Catholic University of Korea, Seoul, Republic of Korea; 3 Sanofi, Seoul, Republic of Korea; 4 NDnex, Saebitgongwon-ro 67, Gwangmyeong-si, Gyeonggi-do 14348 Republic of Korea

**Keywords:** Asthma, Dupilumab, Cost-effectiveness analysis, Economic evaluation, Quality-adjusted life years

## Abstract

**Background:**

A sub-analysis of the Korean population in the LIBERTY ASTHMA QUEST trial (NCT02414854) revealed that dupilumab effectively treated severe uncontrolled asthma. This study aimed to assess the cost-effectiveness of add-on therapy with dupilumab to background therapy in patients ≥ 12 years of age with uncontrolled severe asthma compared to that of background therapy in South Korea.

**Methods:**

The cost-effectiveness analysis was conducted using a Markov model over a lifetime from the Korean healthcare system perspective. Clinical efficacy and utility weights were obtained from post-hoc analyses of the Korean population in the QUEST trial. Data on the costs and treatment setting of exacerbation in a real-world setting were retrospectively collected using the administrative medical database from a single tertiary hospital.

**Results:**

The base-case results indicated that add-on dupilumab therapy increases costs ($112,924 for add-on dupilumab *versus* $29,545 for background therapy alone). However, add-on dupilumab increased quality-adjusted life years (QALYs, 8.03 *versus* 3.93, respectively), with fewer events of severe exacerbations per patient compared to using the background therapy alone (17.920 versus 19.911, respectively). The incremental cost-effectiveness ratio was $20,325 per QALY. Various sensitivity analyses supported the robustness of the base-case results. Probabilistic sensitivity analysis showed that the probability of add-on dupilumab being cost-effective was 87% at a threshold willingness-to-pay of $26,718 (KRW 35 million) per QALY gained.

**Conclusions:**

Dupilumab is cost-effective for adolescents and adults with uncontrolled severe asthma in South Korea. Our study provides evidence to support clinicians and policymakers in making informed decisions for severe asthma management.

**Supplementary Information:**

The online version contains supplementary material available at 10.1186/s13561-024-00532-4.

## Background

Asthma is a chronic lung disease defined, clinically, as a reversible airway obstruction that causes wheezing, coughing, and difficulty breathing [1]. The prevalence of asthma (2–8%) in the general population in South Korea has increased steadily over the past decade [2, 3]. Although standard-of-care therapies, including inhaled corticosteroids (ICS), short-acting beta-agonists, and long-acting beta2-agonist (LABA), have demonstrated efficacy for asthma management [4], approximately 20% of asthma patients have uncontrolled severe asthma despite optimal treatment [5].

Dupilumab (Dupixent®) is the first biologic therapy approved for treating uncontrolled severe asthma with type 2 inflammation, including allergic (anti-immunoglobulin [Ig]-E) and eosinophilic (anti-interleukin [IL]-5) phenotypes [6]. Dupilumab targeting the alpha subunit of the IL-4 receptor covers a broad population with type 2 severe asthma, including allergic, eosinophilic, and oral glucocorticoid–dependent asthma [7]. However, other biologics are indicated for specific phenotypes of type 2 severe asthma: omalizumab (Xolair®), which is an anti-Ig-E antibody for the treatment of allergic asthma, and benralizumab (Fasenra®), reslizumab (Cinqair®), and mepolizumab (Nucala®), which are anti-IL-5 antibodies for treating eosinophilic asthma [8, 9]. Based on the clinical benefits demonstrated in phase 3 clinical trials, dupilumab was approved by the Korea Food and Drug Administration in 2020 as an add-on maintenance therapy for uncontrolled severe asthma with type 2 inflammation in adults and adolescents aged ≥ 12 years [5, 10].

In the National Health Insurance system, which provides universal healthcare coverage to the entire Korean population through mandatory social health insurance, it is crucial to allocate limited healthcare resources efficiently as the financial burden on health insurance increases [11]. Economic evaluations of novel pharmaceuticals conducted in the Korean context serve as a basis for making informed decisions regarding reimbursement [12]. Although add-on dupilumab treatment showed a greater reduction of severe exacerbations and improvement of lung function in Korean patients included in a sub-analysis of the LIBERTY ASTHMA QUEST study (NCT02414854) compared to the overall population in the QUEST trial [13], the cost-effectiveness of dupilumab has not been assessed in Korea. Therefore, whether the additional benefits of dupilumab are sufficient to justify its use as an add-on therapy in terms of economic efficiency remains unclear. This study aimed to assess the cost-effectiveness of add-on therapy with dupilumab to background therapy in patients aged ≥ 12 years with uncontrolled severe asthma compared to background therapy alone in South Korea.

## Methods

### Model overview

A Markov model was designed to reflect the course of patients with persistent uncontrolled asthma based on a literature review [14]. Expert opinion was employed to validity the model structure, including the health states and transitions between them, thereby ensuring its clinical relevance and accuracy. The model reflected the chronic symptoms that patients might experience depending on the asthma control level and the recurring symptoms such as exacerbation events. The target population of the model consisted of Korean patients aged ≥ 12 years with uncontrolled severe asthma despite treatment with medium-to-high dose ICS alongside up to two additional controllers (e.g., leukotriene receptor antagonist or LABA). This treatment strategy corresponded with steps 4–5 of the Global Initiative for Asthma 2022 guidelines [15] and was consistent with the treatment step of the populations included in the QUEST study [13]. The efficacy data for dupilumab were mainly obtained from post-hoc analyses of the QUEST trial for the Korean population [13]. The treatment costs of severe asthma and setting of exacerbation treatment in real-world settings were retrospectively examined at a single tertiary hospital. Based on the European Respiratory Society/American Thoracic Society definition, 59 Korean patients diagnosed with severe asthma [16], who had not previously used biologics, were recruited between November 26, 2015, and March 8, 2023, at the Catholic University of Korea Seoul St. Mary’s Hospital. Data on costs and treatment settings were obtained from the hospital’s administrative medical database. The study was approved by the Institutional Review Board of the Catholic University of Korea Seoul St. Mary’s Hospital (approval No. 2022-2850-0001).

### Model structure

**Fig. 1:**
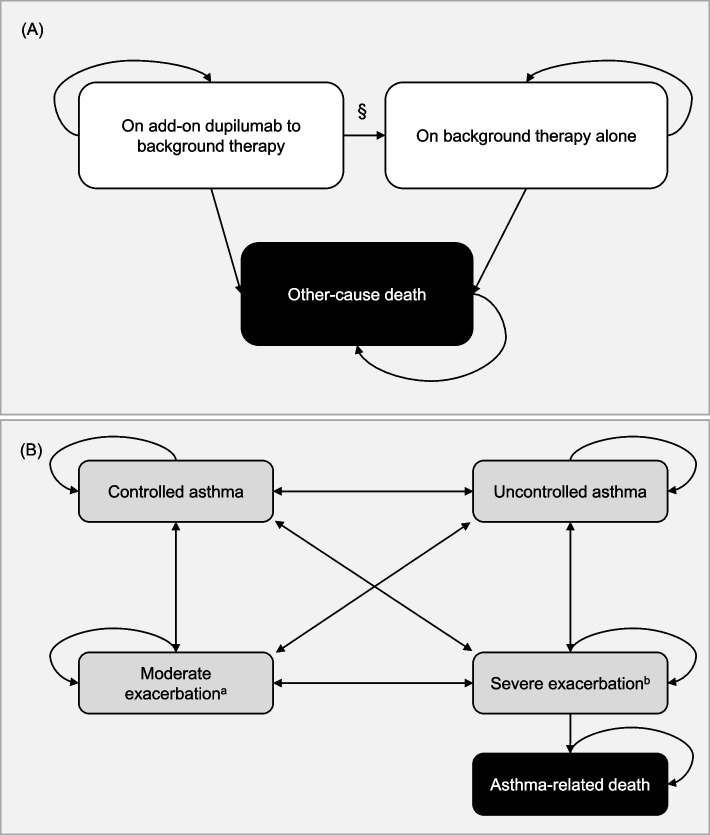
Model structure. **A** Treatment-related health states and other-cause death. §Patients receiving the add-on dupilumab therapy were separated into responders and non-responders after an initial response assessment at 52 weeks. Responders remained in the “add-on dupilumab” state after the assessment, whereas non-responders were assumed to discontinue the add-on dupilumab therapy and transit to the “background therapy alone” state. **B** Disease-related health states, as sub-states within each treatment-related health state. ^a^ “Moderate exacerbation” state was defined based on the loss of asthma control events, excluding severe exacerbation events as collected in the QUEST study. ^b^ “Severe exacerbation” state was defined based on worsening asthma leading to systemic glucocorticoid use for at least 3 days or hospitalization or an emergency department visit requiring treatment using systemic glucocorticoids, as collected in the QUEST study

The Markov health states consist of treatment-related health states (Fig. 1A) and its sub-states, disease-related health states (Fig. 1B). To reflect different transition risks between the treatments and the impact of discontinuing add-on dupilumab, the model separated treatment-related health states into “background therapy alone” state and add-on treatment with dupilumab to background therapy, the “add-on dupilumab” state. All patients received add-on dupilumab or background therapy alone during the first 52 weeks. The patients receiving the add-on dupilumab therapy were classified into responders and non-responders after assessment of the initial response at week 52. Response to dupilumab was defined as at least a 50% reduction in the annualized rate of severe asthma exacerbation events during the treatment period (~ 52 weeks) compared to that in the preceding year. Responders remained in the “add-on dupilumab” state after the assessment, whereas non-responders were assumed to discontinue the therapy and move to the “background therapy alone” state. In addition, patients who discontinued the add-on therapy due to loss of efficacy or adverse events moved to the “background therapy alone” state and could not return to receiving add-on dupilumab. Patients who received background therapy alone at the model start were assumed to remain on it until the end of the time horizon or death, whichever occurred first. Each patient group with one of these two treatment-related health states could experience five mutually exclusive disease-related substates, defined by the asthma control level and occurrence of exacerbations: controlled asthma, uncontrolled asthma, moderate exacerbation, severe exacerbation, and asthma-related death. The “uncontrolled asthma” state was defined as a score of ≥ 1.5 on the Asthma Control Questionnaire 5-question version (ACQ-5), with the “controlled asthma” state defined as an ACQ-5 score of < 1.5 [17]. The “moderate exacerbation” state was defined based on the loss of asthma control events as collected in the QUEST study, excluding severe exacerbation events [5]. The “severe exacerbation” state was further subdivided into a worsening of asthma leading to systemic glucocorticoid use for at least 3 days, hospitalization, or an emergency department (ED) visit requiring systemic glucocorticoid treatment.

All patients in the “add-on dupilumab” or “background therapy alone” groups entered the model with the “uncontrolled asthma” substate. The cycle length for the model was 4 weeks, corresponding to the frequency of reporting exacerbations in the QUEST study. Patients could transit to the “other-cause death” state from any treatment-related health state based on the life table for the general Korean population. Additionally, from the “severe exacerbation” state, patients could transit to the “asthma-related death” state. The death state is an absorbing state, indicating that once a patient enters this state, they cannot transition to any other health state.

### Clinical inputs

Data from the Korean population enrolled in the phase-3 QUEST trial were used to estimate the clinical input [13]. The mean age of the patients (*n* = 74) was 51.9 years—the starting age in the model. Table 1 and Additional file 1: Table S1 provides detailed descriptions of the model inputs.

**Table 1:** Model inputs and variances: base-case and sensitivity analyses

Parameter	Value in the base case	Value in DSA, lower/upper^a^	PSA distribution used	Sources
**Analysis setting**				
Start age (year)	51.9	40.1–63.7	Normal	Mean age in the Korean population from QUEST trial [13]
Time horizon	Lifetime			
**Response and treatment persistence**				
Proportion of responders for dupilumab	0.857	0.686–1.000	Beta	Post-hoc analyses of QUEST trial for the Korean population [13]
Annual long-term discontinuation rates for dupilumab	0.107	0.089/0.125	Gamma	Post-hoc analyses of QUEST trial for ITT population [5]
**Transition probability**				
Transition probabilities between disease-related sub-states	Detailed in Additional file 1: Table S1		Dirichlet	Post-hoc analyses of QUEST trial for the Korean population [13]
Relative effect of experiencing a severe exacerbation beyond the trial period for dupilumab	1.350	1.155–1.545	Log-normal	Published data [14]
Fatality rate for severe exacerbation	0.049	0.039–0.058^b^	Beta	Published data using national health insurance claims data by HIRA [18]
**Proportions of the exacerbation treatment settings**			Dirichlet	Administrative medical database from the hospital
Office visit	0.524			
ED visit	0.143	0.114, 0.171^b^		
Hospitalization	0.333	0.267, 0.400^b^		
**Utility weights**				
** Utility for control-related states without exacerbation**			Beta	Post-hoc analyses of QUEST trial for the Korean population [13]
Controlled asthma	0.907	0.811/1		
Uncontrolled asthma	0.795	0.653/0.937		
** Utility increment for treatment-related states**			Normal	Post-hoc analyses of QUEST trial for the Korean population [13]
Dupilumab (all patients)	+ 0.023	-0.100/0.146		
Dupilumab (responders)	+ 0.039	-0.083/0.161		
** Disutility for exacerbation-related states**^**c**^			Normal	Post-hoc analyses of the QUEST trial for the ITT population [5]
Moderate exacerbation	-0.125	-0.135/-0.115		
Severe exacerbation – Office visit	-0.161	-0.176/-0.146		
Severe exacerbation – ED visit	-0.164	-0.290/-0.038		
Severe exacerbation – Hospitalization	-0.186	-0.311/-0.061		
**Duration with exacerbations (days)**			Gamma	Post-hoc analyses of QUEST trial for the ITT population [5]
** For “Add-on dupilumab”**				
Moderate exacerbation	17.2	16.4/18.0		
Severe exacerbation – Office visit	14.4	13.7/15.1		
Severe exacerbation – ED visit	13.2	10.0/16.4		
Severe exacerbation – Hospitalization	18.5	16.0/21.1		
** For “Background therapy alone”**				
Moderate exacerbation	16.6	16.1/17.1		
Severe exacerbation – Office visit	18.0	17.2/18.7		
Severe exacerbation – ED visit	20.2	17.4/23.0		
Severe exacerbation – Hospitalization	28.0	22.0/34.1		

*CI* Confidence interval, *DSA* Deterministic sensitivity analysis, *ED* Emergency department, *HIRA* Health Insurance Review and Assessment Service, *PSA* Probabilistic sensitivity analysis, *SE* Standard error

^a^In DSA, the parameters were varied using 95% CIs or SE based on the data sources

^b^The source for parameter values did not report SEs or CIs; therefore, it was assumed that the SE was equivalent to 20% of the mean

^c^With the disutility approach, the utility associated with exacerbations was determined by applying a disutility to the utility value in the “controlled asthma” state

The proportion of responders to add-on dupilumab was obtained from the sub-analyses of the QUEST trial [13]. Based on the proportion of non-responders, a transition probability from the “add-on dupilumab” state to the “background therapy alone” state was calculated. To consider the persistence of dupilumab treatment after the initial response assessment, the long-term discontinuation rates obtained from the QUEST trial [5] were applied to add-on dupilumab responders (Table 1).

In the model, the “severe exacerbation” state was categorized based on the trial’s definitions, using the treatment setting: exacerbations leading to office visits, ED visits, or hospitalization with worsening asthma leading to systemic glucocorticoid use for at least 3 days [5]. The proportion of patients treated in each treatment setting was retrospectively evaluated using administrative medical data from a single center (Table 1).

Transition probabilities between disease-related health states were estimated based on post-hoc analyses of the QUEST trial (Additional file 1: Table S1) [13]. The transition probabilities were divided into 0–12 weeks, 12–52 weeks, and post 52 weeks probabilities and applied to the model. The QUEST trial revealed differences in asthma control risk before and after the initial lung function assessment at 12 weeks. Therefore, separate transition probabilities were applied for the 0–12 weeks of the trial and beyond 12 weeks to reflect the variation in transition probabilities over time. Only responders remained in the “add-on dupilumab” state after the 52-week assessment; therefore, the transition probabilities estimated for responders to dupilumab during the 12–52 weeks were applied beyond 52 weeks. In addition, to account for the increased risk of severe exacerbations beyond the follow-up period of the trial (i.e., after 52 weeks), a factor of 1.35 was assumed for transition probabilities. This factor was based on the assumption that severe exacerbation rates in a real-world setting would be higher than those observed in a clinical trial setting due to improved monitoring and restricted inclusion criteria [14].

All-cause and asthma-related mortality rates in the general population were derived from the cause-of-death statistics provided by the Korean Statistical Office﻿ [19]. The annual mortality rates for other-cause deaths were calculated by subtracting the number of asthma-related deaths from the number of all-cause deaths. Based on a study that reported the 28-day case fatality rate after exacerbation with severe asthma using the National Patient Sample in South Korea [18], the fatality rate after severe exacerbation was 4.9% and was applied in the model irrespective of the treatment setting (Table 1).

### Utility weights

Utility weights associated with “controlled asthma” and “uncontrolled asthma” states were obtained using post-hoc analyses of the EQ-5D values among the Korean population in the QUEST trial [13], using the Korean tariff [20]. As utility differences were observed between patients treated with add-on dupilumab (particularly responders) and those treated with background therapy alone, a utility increment for add-on dupilumab was applied to adjust for variation based on the treatment. The disutilities associated with exacerbations were derived from the QUEST trial (Table 1). The utilities in the “moderate or severe exacerbation” states were determined by adding disutility to the utility value in the “controlled asthma” state.

### Costs

Dupilumab costs included drug acquisition costs obtained from the drug price list provided by the Health Insurance Review and Assessment Service (HIRA) and drug administration costs obtained from the resource use-based unit cost provided by the HIRA [21] (Table 2). The dosage regimen for dupilumab, 200 mg (initial dose of 400 mg) or 300 mg (initial dose of 600 mg) every 2 weeks, was based on the QUEST trial [13]. The cost of the health states associated with asthma control and exacerbation was estimated using administrative medical data from the Seoul St. Mary’s Hospital and applied equally to both treatment arms. The costs for each health state consisted of expenditures on diagnosis, pharmacological treatments, and resources used for in-patient and out-patient services. Pharmacological treatment costs included drug costs for background therapy, which reflected the proportion of controller medications used in real-world settings in Korea. All costs were expressed in February 2023 United States dollars (USD), using an exchange rate of 1 USD to 1310 Korean Won (KRW), and adjusted for inflation, where applicable, using the consumer price index for healthcare.

**Table 2:** Cost inputs

Parameter	Value in the base case	Value in DSA, lower/upper^a^	PSA distribution used	Sources
Drug cost per cycle (USD)			Gamma	List price of dupilumab 200 mg/300 mg by HIRA
Dupilumab per the first cycle	1064	851/1277		
Dupilumab per subsequent cycles	2129	1703/2555		
Health states cost,^b^ per cycle (USD)			Gamma	Administrative medical database from the hospital
Controlled asthma	44	36/53		
Uncontrolled asthma	106	85/128		
Moderate exacerbation	191	152/229		
Severe exacerbation – Office visit	141	113/169		
Severe exacerbation – ED visit	300	240/360		
Severe exacerbation – Hospitalization	4324	3460/5189		

All costs are expressed in 2023 USD using an exchange rate of 1 USD to 1310 KRW

The model cycle length was 4 weeks

*DSA* Deterministic sensitivity analysis, *ED* Emergency department, *HIRA* Health Insurance Review and Assessment Service, *KRW* Korean Won, *PSA* Probabilistic sensitivity analysis, *USD* United Stated dollar

^a^Drug costs and health states cost varied by ± 20%

^b^The costs for each health state consisted of expenditures on diagnosis, pharmacological treatments, and resources used for in-patient and out-patient services

### Base case analysis

A cost-utility analysis was conducted over the lifetime. The incremental cost-effectiveness ratio (ICER) of add-on dupilumab compared to background therapy alone was presented, which was calculated by dividing the incremental cost by the additional quality-adjusted life-years (QALYs) gained. This study, conducted from the perspective of the Korean healthcare system, included direct medical costs incurred within the healthcare system, while excluding transportation costs, time costs, and productivity losses. And a 4.5% annual discount rate for cost and QALYs were used with half-cycle corrections, based on the recommendations of the Korean guidelines for pharmacoeconomic evaluation [22]. The model was programmed in Microsoft Excel with macro programming supported by Visual Basic for Applications.

### Scenario analyses

Structural uncertainty regarding the model assumptions was assessed through several scenario analyses, as follows: Scenario 1, 0% discount rate for costs and QALY; Scenario 2, 3.0% discount rate for costs and QALY; Scenario 3, time horizon of 10 years; Scenario 4, time horizon of 20 years; Scenario 5, no adjustment for the long-term risk of experiencing severe exacerbations based on observed trial data; Scenario 6, the fatality rate for exacerbation requiring office or ED visits assumed to be equal to all-cause mortality of the general population; Scenario 7, utility values from previous literature [23, 24] were applied as shown in Additional file 1: Table S2; and Scenario 8, analysis from the societal perspective including the productivity loss costs due to morbidity and premature death, with the detailed methods presented in Additional file 2: Supplementary Methods.

### Sensitivity analyses

Deterministic sensitivity analyses (DSA) and probabilistic sensitivity analyses (PSA) were conducted to assess parameter uncertainty. In DSA, the impact of a variation in transition probabilities, exacerbation treatment setting, utility weights, and costs related to disease control and exacerbations were examined. PSA was performed using a second-order Monte Carlo simulation with 1000 iterations, in which, each parameter estimate was sampled from its distribution. The Dirichlet distribution was assigned to the proportions of the setting of exacerbation treatment and transition probabilities. The gamma distribution was applied to costs, duration of exacerbation, and discontinuation rates. The utilities and proportion of responders were sampled from the beta distribution. The relative effect of experiencing severe exacerbation beyond the trial period for dupilumab was sampled from the log-normal distribution. Regarding the starting age, the normal distribution was assumed (Table 1). The PSA results were presented as a cost-effectiveness plane and cost-effectiveness acceptability curve (CEAC) using a willingness-to-pay (WTP) threshold of $26,718 (KRW 35 million) per QALY [25].

## Results

### Base-case analysis

The number of severe exacerbations per patient over a lifetime was 17.920 and 19.911 in the dupilumab add-on and background therapy alone groups, respectively, with 1.992 fewer events in the dupilumab group lifetime compared to background therapy alone group. Cost-utility analysis indicated that add-on dupilumab was more effective than background therapy alone (8.03 QALYs *versus* 3.93 QALYs, respectively) but was more expensive ($112,924 *versus* $29,545, respectively). Add-on dupilumab had incremental costs of $83,379 and incremental QALYs of 4.10 compared to background therapy alone over the lifetime horizon for patients with uncontrolled severe asthma. Through the base-case analysis, ICER was calculated as $20,325 per QALY gained (Table 3).

**Table 3:** Results of base-case analysis

	**Add-on dupilumab to background therapy**	**Background therapy alone**	**Incremental**
Number of exacerbations	36.341	31.636	4.705
Moderate exacerbations	18.421	11.725	6.697
Severe exacerbations	17.920	19.911	-1.992
Requiring office visit	5.088	5.654	-0.566
Requiring ED visit	1.438	1.598	-0.160
Requiring hospitalization	11.393	12.660	-1.266
Number of deaths	0.999	1.000	-0.001
Exacerbation-related deaths	0.871	0.968	-0.097
Non-asthma deaths	0.128	0.032	0.096
Drug costs^a^ (USD)	87,333	0	87,333
Disease management costs (USD)	5696	2716	2,980
Exacerbation-related costs (USD)	19,895	26,829	-6,934
Productivity loss costs^b^ (USD)	6317	16,851	-10,534
Due to morbidity	5378	15,173	-9,795
Due to premature death	939	1678	-739
Total costs (USD)^c^	112,924	29,545	83,379
Total effectiveness (QALY gained)	8.03	3.93	4.10
ICER (USD/QALY)			20,325

All costs are expressed in 2023 USD using an exchange rate of 1 USD to 1310 KRW

*ED* Emergency department, *ICER* Incremental cost-effectiveness ratio, *KRW* Korean Won, *QALY* Quality-adjusted life-year, *USD* United States dollar

^a^Drug costs included drug acquisition costs and drug administration costs

^b^Productivity loss costs were only considered in the scenario analysis from a societal perspective

^c^Productivity loss costs were not included

### Scenario analyses

Applying 0% and 3% discount rates in the scenario analysis yielded a –9.9% ($18,303/QALY) and –3.4% ($19,640/QALY) decrease in the ICER, respectively, compared with the ICER from the base-case analysis (Table 4). As the discount rate decreased, the additional QALY gains for dupilumab increased significantly. In the 10- and 20-year time horizons, the ICER increased by 21.2% ($24,625/QALY) and 3.0% ($20,935/QALY), respectively. With a decreasing time horizon, the additional QALY gains for dupilumab decreased significantly. Incremental QALYs were most influenced by the discount rate and time horizon. In the scenario that applied the long-term risk of experiencing severe exacerbations based on observed data, incremental costs were similar to that observed in the base-case analysis. However, additional QALY gains for dupilumab decreased, leading to an ICER increase of 10.1% ($22,386/QALY). The scenario analysis of applying all-cause mortality of the general population to the fatality rate for exacerbation requiring office or ED visits yielded a 23.0% ($24,999/QALY) increase in ICER. In the scenario analysis using the societal perspective, an additional cost of $10,534 per patient for productivity loss, including $9795 due to morbidity and $739 due to premature death, were incurred for the background therapy alone arm, with this scenario yielding a 12.6% decrease in the ICER ($17,757/QALY) (Table 4).

**Table 4:** Cost-utility results in scenario analyses

	**Intervention**	**Cost (USD)**	**Incremental Cost (USD)**	**QALYs**	**Incremental QALYs**	**ICER (USD/QALY)**
Discount rate of 0%	Add-on dupilumab to background therapy	154,798	118,232	11.24	6.46	18,303
	Background therapy alone	36,566		4.78		
Discount rate of 3%	Add-on dupilumab to background therapy	124,017	92,487	8.88	4.71	19,640
	Background therapy alone	31,530		4.17		
Time horizon 10 years	Add-on dupilumab to background therapy	86,595	60,554	5.97	2.46	24,625
	Background therapy alone	26,041		3.51		
Time horizon 20 years	Add-on dupilumab to background therapy	107,913	78,715	7.65	3.76	20,935
	Background therapy alone	29,198		3.89		
Long-term risk of experiencing severe exacerbations based on observed trial data without adjustment	Add-on dupilumab to background therapy	112,040	83,664	9.08	3.74	22,386
	Background therapy alone	28,376		5.35		
Fatality rate for exacerbation requiring office or ED visit: assumed to be equal to all-cause mortality of the general population	Add-on dupilumab to background therapy	126,864	74,533	9.60	2.98	24,999
	Background therapy alone	52,332		6.62		
Utility weights: applied based on published literature	Add-on dupilumab to background therapy	112,924	83,379	7.94	4.24	19,674
	Background therapy alone	29,545		3.71		
Includes the productivity loss costs from a societal perspective	Add-on dupilumab to background therapy	119,240	72,845	8.03	4.10	17,757
	Background therapy alone	46,396		3.93		

All costs are expressed in 2023 USD using an exchange rate of 1 USD to 1310 KRW

*ED* Emergency department, *ICER* Incremental cost-effectiveness ratio, *KRW* Korean Won, *QALY* Quality-adjusted life-year, *USD* United States dollar

### Sensitivity analyses

**Fig. 2:**
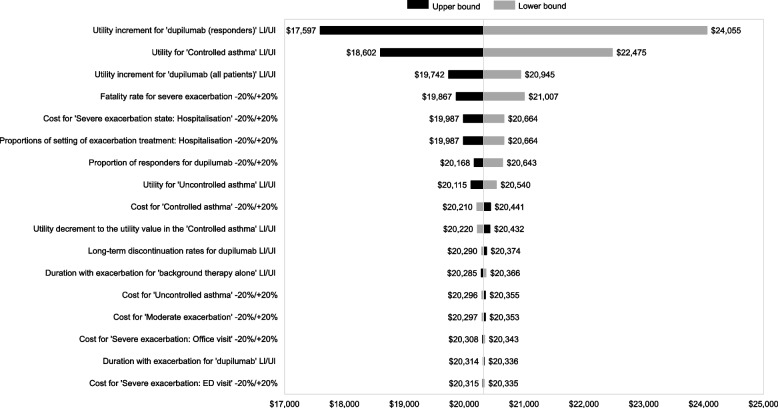
Tornado diagram for add-on dupilumab therapy versus background therapy alone. Ll: Lower limit; Ul: Upper limit; ED: emergency department

The tornado diagram indicated that the most influential parameters on ICER were utility increment for dupilumab responders and utility for a “controlled asthma” state. However, the model was not sufficiently sensitive to change the results for any one parameter. Add-on dupilumab was the cost-effective strategy in all variations tested in the DSA (Fig. 2).

**Fig. 3:**
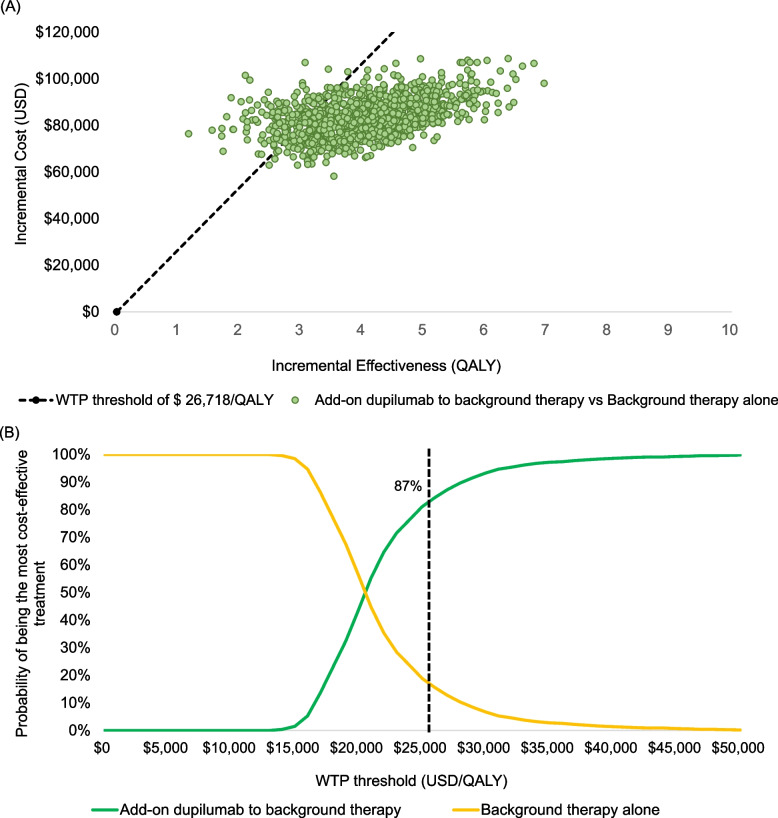
Results of probabilistic sensitivity analysis. **A** Cost-effectiveness plane. **B** Cost-effectiveness acceptability curve. The dotted line indicates the WTP threshold. QALY: quality-adjusted life-year; WTP: willingness-to-pay

PSA results are presented in the cost-effectiveness planes showing the distributions of incremental costs and effects between the two arms (Fig. 3A). Most values were distributed below the cost-effectiveness threshold in the northeastern quadrant, indicating that the base-case results were robust. The CEAC (Fig. 3B) showed that the probability of add-on dupilumab being cost-effective compared with background therapy alone was approximately 87% at a WTP threshold of $26,718 per QALY gained.

## Discussion

This study evaluated the cost-effectiveness of add-on dupilumab compared to background therapy alone in patients with uncontrolled severe asthma using clinical data from the Korean QUEST trial and real-world data from the hospital’s administrative medical database. By incorporating local data into the model, we demonstrated that add-on dupilumab was cost-effective compared with background therapy alone, which can be generalized to all patients with severe asthma in South Korea. Our findings indicated that if 1000 patients were treated with dupilumab, compared to background therapy alone, approximately 2000 events of severe exacerbation could be averted over a lifetime. In addition, all sensitivity analyses supported the robustness of the cost-effectiveness of add-on dupilumab. Therefore, our study provides real-world evidence that can support clinicians and policymakers in making informed decisions regarding patient access to dupilumab to manage severe uncontrolled asthma in South Korea.

Two previous studies have assessed the cost-effectiveness of dupilumab in uncontrolled severe asthma patients [14, 26]. A cost-utility analysis comparing add-on dupilumab to standard therapy alone was reported in Colombia [26]; however, it is difficult and infeasible to transfer the economic evaluation results across countries without any correction [27]. In their economic evaluation based on values obtained from several papers, dupilumab was not cost-effective with the estimated ICERs of $50,160 per QALY gained, which is contrary to our results based on context-specific epidemiological, clinical outcome, resource utilization, cost, and quality of life data in Korea. Tohda et al. [14] evaluated the cost-effectiveness of dupilumab in a population with uncontrolled severe asthma using a Markov model similar to ours, but there are some notable differences between their study and ours. They focused on a subgroup of patients with oral corticosteroid-dependent severe asthma, assessing the cost-effectiveness of dupilumab compared to other biologics (such as benralizumab, mepolizumab, and omalizumab), targeting specific phenotypes of type 2 severe asthma. The efficacy of dupilumab and other biologics has been established through indirect comparisons, due to the absence of head-to-head clinical trials. In contrast, our study evaluated the cost-effectiveness of dupilumab in a broader population of type 2 asthma, based on the head-to-head QUEST trial. Given the different target populations, and limitations in patient accessibility under domestic reimbursement conditions, other biologics cannot be a complete alternative to dupilumab. Therefore, they were not included as comparators in our study.

The QUEST sub-analysis study [13] used to estimate treatment efficacy in our study indicated that add-on dupilumab therapy was effective in Korean patients with severe asthma. The magnitude of risk reduction for severe exacerbation and improvements in the forced expiratory volume in one second associated with moderate exacerbation improvement was much higher in Korean patients than in the overall patients of the QUEST data [13]. In addition, Korean patients treated with dupilumab had a greater improvement in the risk of severe exacerbation than Japanese patients treated with dupilumab, with a higher proportion of responders to dupilumab (85.7% for the Korean population in our study *versus* 73.7% for the Japanese population) [14]. These variabilities in dupilumab efficacy might be explained by ethnicity or treatment compliance [28].

Considering the difficulties in observing asthma-related deaths after severe exacerbation in clinical trials, we applied the 28-day case fatality related to severe exacerbation among Korean patients with severe asthma reported in a previous study [18]. In the previous study, the fatality rate within 28 days after severe asthma exacerbation requiring hospital admission was 4.9%. As the fatality rate associated with exacerbations leading to an ED or office visit was not reported, the fatality rate after severe exacerbation was equally applied in the base case regardless of the treatment setting. However, as asthma-related mortality is heterogeneous, at 0.43–9.8% across treatment settings in other countries [29–31], we conducted a scenario analysis adapting a conservative assumption that the fatality rate after non-hospitalized exacerbations is the same as the mortality rate in the general population, showing a slight increase in ICER.

The incremental costs were mainly driven by the drug acquisition cost of dupilumab, which has been reimbursed and managed under a risk-sharing agreement since 2020 in South Korea. Risk-sharing agreements distribute the responsibility for the risk that involves uncertainty regarding the clinical outcomes, cost-effectiveness, and financial impact on health insurance of new drugs between payers and pharmaceutical manufacturers. This enables payers to maintain reimbursement decision principles that consider these factors, and manage the uncertainties on effectiveness and budget [32]. Although the agreement details were unavailable due to confidentiality, incorporating a refund or discount on drug price into the model could reduce medical costs for the add-on dupilumab group in economic evaluations conducted from the healthcare system perspective, thereby suggesting the expectation of better cost-effectiveness results for this group.

A scenario analysis from the societal perspective that considered the additional burden of productivity loss due to hospitalization and premature death showed that the costs of productivity loss for a patient with severe asthma treated with background therapy alone accounted for 36.3% ($16,851/$46,396) of total expected costs. This value was slightly higher than that observed in previous studies, which reported that the cost of productivity loss due to morbidity and mortality in Korea accounted for 15.1–27.0% of the total socioeconomic cost [33, 34]. Patients with severe asthma tend to experience more frequent events of asthma exacerbations and prolonged hospitalizations [3]. Therefore, it is reasonable that the estimates of productivity loss costs for severe patients in our study were higher than those for all patients with asthma, regardless of severity in previous studies. The scenario results showed that the productivity loss costs incurred in the background therapy alone group were higher than those in the add-on dupilumab group, increasing the cost-effectiveness of dupilumab.

Our study has some limitations. First, while the estimates of clinical efficacy were based on data from clinical trials with a 52-week intervention period and a 12-week follow-up period after the intervention [13], we assumed that the efficacy would be maintained during the lifetime horizon of the model. Therefore, after the initial response assessment, responders continued to use the add-on dupilumab until they discontinued treatment due to adverse events or noncompliance. An open-label extension study confirmed that the efficacy of dupilumab in adult patients with severe asthma was sustained for 148 weeks, supporting the long-term use of dupilumab [35]. Second, the study was designed to assess the cost-effectiveness of add-on dupilumab in the Korean population using inputs from the Korean setting; however, some local data on discontinuation rates and disutility for exacerbation-related states were unavailable. Thus, we used data from the entire QUEST population for these parameters; however, this uncertainty had a minimal impact on the base-case results, according to DSA. Third, we used cost data for 59 patients from a single tertiary hospital; therefore, the estimated costs might have been biased owing to an unrepresentative small sample and selection bias. However, previous studies using representative national health insurance claims data from the HIRA to estimate asthma control and exacerbation costs reported results similar to ours: patients with severe exacerbations, especially those leading to hospitalization, incurred enormous costs [18, 36]. Despite these limitations, the various sensitivity and scenario analyses confirmed that our model was robust.

## Conclusion

From a Korean healthcare system perspective, add-on dupilumab to background therapy is a cost-effective option over a lifetime horizon for patients with uncontrolled severe asthma compared to background therapy alone. Our study provides evidence for clinicians and policymakers to make informed decisions.

### Supplementary Information

Additional file 1: Table S1. Transition probabilities. Table S2. Utility weights used in scenario analyses.

Additional file 2: Supplementary Methods. Productivity loss costs due to morbidity and premature death.

## Data Availability

No datasets were generated or analysed during the current study.

## Abbreviations

ACQ-5: Asthma Control Questionnaire 5-question version
CEAC: Cost-effectiveness acceptability curve
DSA: Deterministic sensitivity analysis
ED: Emergency department
HIRA: Health Insurance Review and Assessment Service
ICER: Incremental cost-effectiveness ratio
ICS: Inhaled corticosteroids
Ig: Immunoglobulin
IL: Interleukin
KRW: Korean Won
LABA: Long-acting beta2-agonist
PSA: Probabilistic sensitivity analysis
QALY: Quality-adjusted life-year
WTP: Willingness-to-pay
USD: United States dollar
