# Supplementary material for: Cost-effectiveness analysis of dupilumab among patients with uncontrolled severe asthma using LIBERTY ASTHMA QUEST Korean data
Source: Health Econ Rev. 2024 Aug 26;14:67. doi: 10.1186/s13561-024-00532-4 (PMC11346198; doi:10.1186/s13561-024-00532-4)
Supplement: Supplementary file 1 — Additional file 1: Table S1. Transition probabilities. Table S2. Utility weights used in scenario analyses. [file 13561_2024_532_MOESM1_ESM.docx]

Table S1. Transition probabilities

| **Health states** | **To** | | | | | | | |
| --- | --- | --- | --- | --- | --- | --- | --- | --- |
|  | **Controlled asthma** | **Uncontrolled asthma** | **Moderate exacerbation** | **Severe exacerbation** | **Controlled asthma** | **Uncontrolled asthma** | **Moderate exacerbation** | **Severe exacerbation** |
| **From** | **Background therapy alone** | | | | **Add-on dupilumab to background therapy** | | | |
| 0–12 weeks | | | | | | | | |
| Controlled Asthma | 75.69% | 4.45% | 11.03% | 8.82% | 88.92% | 7.12% | 2.38% | 1.59% |
| Uncontrolled Asthma | 24.58% | 36.87% | 10.84% | 27.71% | 40.32% | 44.93% | 10.26% | 4.49% |
| Moderate Exacerbation | 42.79% | 10.70% | 41.86% | 4.65% | 29.63% | 14.81% | 53.33% | 2.22% |
| Severe Exacerbation | 33.60% | 22.40% | 8.00% | 36.00% | 80.00% | 0.00% | 6.67% | 13.33% |
| 12–52 weeks | | | | | | | | |
| Controlled Asthma | 73.10% | 7.05% | 11.03% | 8.82% | 91.06% | 4.97% | 2.38% | 1.59% |
| Uncontrolled Asthma | 21.27% | 40.18% | 10.84% | 27.71% | 26.01% | 59.25% | 10.26% | 4.49% |
| Moderate Exacerbation | 29.71% | 23.77% | 41.86% | 4.65% | 19.05% | 25.39% | 53.33% | 2.22% |
| Severe Exacerbation | 24.35% | 31.65% | 8.00% | 36.00% | 36.36% | 43.64% | 6.67% | 13.33% |
| 52+ weeks | | | | | | | | |
| Controlled Asthma | 70.01% | 7.05% | 11.03% | 11.91% | 93.70% | 4.12% | 1.72% | 0.46% |
| Uncontrolled Asthma | 11.57% | 40.18% | 10.84% | 37.41% | 25.45% | 60.00% | 14.55% | 0.00% |
| Moderate Exacerbation | 28.09% | 23.77% | 41.86% | 6.28% | 16.67% | 23.33% | 60.00% | 0.00% |
| Severe Exacerbation | 11.75% | 31.65% | 8.00% | 48.60% | 100.00% | 0.00% | 0.00% | 0.00% |
| Source: Calculated from post-hoc analyses of QUEST trial for Korean population [1] | | | | | | | | |

Table S2. Utility weights used in scenario analyses

| **Health state** | **Value** | **95 % confidence interval** | **Sources** |
| --- | --- | --- | --- |
| **Utility for control-related states without exacerbation** |  |  |  |
| Controlled asthma | 0.937 | 0.923–0.952 | [2] |
| Uncontrolled asthma | 0.728 | 0.707–0.749 |  |
| Moderate exacerbation | 0.649 | 0.628–0.670 |  |
| Severe exacerbation-Office visit | 0.570 | 0.549–0.591 | [3] |
| Severe exacerbation-ED visit | 0.570 | 0.549–0.591 |  |
| Severe exacerbation-Hospitalization | 0.330 | 0.309–0.351 |  |

ED = emergency department.

**References**

1. Rhee CK, Park JW, Park HW, Cho YS. Effect of dupilumab in Korean patients with uncontrolled moderate-to-severe asthma: A Liberty ASTHMA QUEST sub-analysis. Allergy Asthma Immunol Res. 2022;14:182–95.

2. Willson J, Bateman ED, Pavord I, Lloyd A, Krivasi T, Esser D. Cost effectiveness of tiotropium in patients with asthma poorly controlled on inhaled glucocorticosteroids and long-acting beta-agonists. Appl Health Econ Health Policy. 2014;12:447–59.

3. Lloyd A, Price D, Brown R. The impact of asthma exacerbations on health-related quality of life in moderate to severe asthma patients in the UK. Prim Care Respir J. 2007;16:22–7.
