# Supplementary material for: Cost-effectiveness analysis of dupilumab among patients with uncontrolled severe asthma using LIBERTY ASTHMA QUEST Korean data
Source: Health Econ Rev. 2024 Aug 26;14:67. doi: 10.1186/s13561-024-00532-4 (PMC11346198; doi:10.1186/s13561-024-00532-4)
Supplement: Supplementary file 2 — Additional file 2: Supplementary Methods. Productivity loss costs due to morbidity and premature death. [file 13561_2024_532_MOESM2_ESM.docx]

**Supplementary Methods**

**Productivity loss costs due to morbidity and premature death**

In our study, the cost of productivity loss due to morbidity is the opportunity cost of time lost due to hospitalization in patients with severe asthma exacerbation. Based on the human capital approach, which estimates lost productivity as the expected earnings lost due to disease [1], the productivity loss cost due to morbidity was calculated per cycle length by multiplying the number of patients hospitalized by the length of hospital stay per patient, age-specific average daily income [2], and employment rates for those aged 20–65 years [3]. The cost of productivity loss due to premature mortality was estimated as the loss of potential earnings until the age of 65 years because of premature death caused by severe asthma. It was calculated per cycle by multiplying the number of asthma-related deaths obtained from the model by the age-specific average income and employment rates for each deceased person. A discount rate of 4.5% was applied to reflect the present value of the future income. Average daily income was derived from the age- and sex-specific average annual income provided by the Korean Statistical Information Service [2]. The total productivity loss costs added to the background therapy alone arm were calculated as the sum of the cost differences between the two arms over a lifetime horizon.

Productivity loss costs were calculated using the following equations:

$$\text{Productivity loss cost due to morbidity per cycle}$$

$$\text{ }\text{= }Number of patients with severe exacerbation leading to hospitalization\times Length of hospital stay in patients with severe asthma exacerbation$$

$$\times Average daily income by age \times Employment rates of 20-65 years by age$$

$$\text{Productivity loss cost due to premature mortality per cycle }$$

$$\text{= }Number of deaths caused by severe asthma \times Average monthly income by age \times Employment rates of 20-65 years by age$$

**References**

1. Liljas B. How to calculate indirect costs in economic evaluations. Pharmacoeconomics. 1998;1:1-7.
2. Survey on working status 2022. Korean Statistical Information Service. <https://kosis.kr/statHtml/statHtml.do?orgId=118&tblId=DT_118N_LCE0004&conn_path=I2>. Accessed March 30, 2023.
3. Economically active population survey 2022. Korean Statistical Information Service. <https://kosis.kr/statHtml/statHtml.do?orgId=101&tblId=DT_1DA7002S&conn_path=I2>. Accessed March 30, 2023.
